# Paraneoplastic Neurologic Syndromes in Children: A Review Article

**Published:** 2013

**Authors:** Samin ALAVI

**Affiliations:** 1 Pediatric Congenital Hematologic Disorders Research Center, Mofid Children’s Hospital, Shahid Beheshti University of Medical Sciences, Tehran, Iran

**Keywords:** Paraneoplasic neurological syndromes, Unconeural Antibodies, Pediatric cancer

## Abstract

**Objective:**

Paraneoplastic neurological syndromes (PNS) were initially defined as neurological syndromes with unknown etiology that often associate with cancer. This broad definition may lead to misconception that any neurological syndrome, which coincides with a cancer might be considered as PNS. In the last two decades it has been suggested that PNSs are mainly immune-mediated. The detection of onconeural antibodies has been very helpful in indicating the existence of a tumor and defining a given neurological syndrome as paraneoplastic. However, PNS may occur without onconeural antibodies, and the antibodies can occur with no neurological syndrome; thus, their presence should not be the only condition to define a neurological syndrome as paraneoplastic. Diagnosis of paraneoplastic syndromes in children may result in early detection and treatment of the pediatric cancer and can reduce the neurological damage that is the major source of morbidity in children with successfully treated tumors. This study reviews the presenting symptoms, immunology, and management options for paraneoplastic syndromes, focusing on those most commonly reported in children.

## Introduction

The term PNS refers to signs or symptoms that result from damage to organs or tissues that are far from the site of a malignant neoplasm or its metastases. PNSs are much less common than direct, metastatic, and treatment related complications of cancer, but are important because they could cause severe neurological morbidity and mortality and often present to the neurologist in a patient without a known malignancy. Paraneoplastic syndromes can affect most organs and tissues (1). Paraneoplastic syndromes happen because the tumor secretes substances, which mimic normal hormones or which interfere with circulating proteins. Paraneoplastic neurologic disorders are caused by similar mechanisms, such as carcinoid myopathy and encephalopathy (2); however, most of PNS are immune- mediated (3). Obviously, damage to the nervous system by cancer-induced coagulopathies or opportunistic infections are not considered to be paraneoplastic neurologic disorders.

PNSs are rare, and affecting less than 1/10,000 patients with cancer. PNS can affect various parts of the central and peripheral nervous system, the neuromuscular junction, and muscle. They can be isolated or occur in association. Paraneoplastic neurologic disorders are usually severe, often disabling, and sometimes lethal (4). In most of patients, the neurological disorder develops prior to the cancer becomes clinically obvious and the patient is referred to the neurologist who is responsible for identifying a neurological disorder as paraneoplastic (5). In the last two decades, it has been approved that some PNSs are associated with antibodies against antigens that are expressed by both the tumor and the nervous system (onconeural antibodies). Although numerous types of paraneoplastic antibodies have been described (1,6-8), less than half of patients with PNS bear paraneoplastic antibodies (7). Thus, the absence of paraneoplastic antibodies cannot rule out the diagnosis of PNS. Many reports suggest that patients who suffer from paraneoplastic neurologic disorders have a better prognosis than patients with histologically identical tumors that are not associated with paraneoplastic neurologic disorders (9). In November 2002, an international panel of neurologists who were interested in the field of PNS started to establish guidelines to provide more strict diagnostic criteria for PNS. According to their discussion, the panel concluded that the diagnostic criteria of a neurological syndrome as paraneoplastic must be based on the presence or absence of cancer and the definitions of classical versus non- classical syndromes and well characterized onconeural antibody (7).

## Diagnostic criteria for PNS

The panel suggested that there should be two levels of diagnostic evidence for definition of a neurological syndrome as paraneoplastic: “definite” and “possible”. Each level can be reached combining a series of criteria. The panel recognized that the term “possible” can include true PNS, but also the coincidental relationship of two independent disorders (the neurological syndrome and cancer) should also be considered. The panel emphasized that definite and possible PNS have in common the requirement to exclude other known causes that can clarify the neurological syndrome, even if onconeural antibodies are positive (7).

## Criteria for definite PNS

1- A classical neurologic syndrome (according to the syndromes defined in Table 1) and cancer that develops whitin five years of the diagnosis of the neurological disorder. In this setting, the presence of onconeural antibodies is not necessary. The time period of five years has been derived from previous work that revealed in patients with classical syndromes, the tumor is nearly always diagnosed within five years following the onset of the PNS (8,10).

2. A non-classical neurologic syndrome that resolves or significantly improves after chemotherapy without concomitant immunotherapy, provided that the syndrome is not susceptible to spontaneous remission. PNS should not been applied to patients whose treatment of the tumor consisted of drugs that are immunosuppressive and these drugs are known to be able to improve the associated neurological syndrome (7).

3. A non-classical syndrome with onconeural antibodies (well characterized or not) and cancer that develops within five years of the diagnosis of the neurological disorder (6).

4. A neurological syndrome (classical or not) with well characterized onconeural antibodies (anti-Hu,Yo, CV2, Ri, Ma2, or amphiphysin) and no cancer.

This set of criteria may have a very small number of false positive cases that will never develop cancer despite the presence of well characterized onconeural antibodies (Table 2). A conceivable explanation is that the tumor was eliminated by the immune response (1). In contrast, anti-Tr antibodies cannot be used for classification of the PNS as definite in the absence of cancer, because 11% of patients reported that never developed Hodgkin’s disease and the antibody disappeared in the follow-up (11).

## Criteria for possible PNS

1. A classical syndrome, no onconeural antibodies, no cancer but at high risk to have an underlying tumour. Some classical syndromes are not related to onconeural antibodies and may also occur in the absence of cancer. The diagnosis of cancer in the follow-up is the only way to define the syndrome as definite PNS. All these patients should have at least an initial cause for cancer.

If cancer never develops after five years, the syndrome should not be included in possible PNS category. There are some clues that can assist neurologists to predict which patients with these classical syndromes are at high risk for an underlying cancer. The panel’s recommendation is that the diagnosis of possible PNS in the setting of the present criteria should apply only to those classical syndromes that also have the identified risks for an underlying tumor.

2. A neurological syndrome (classical or not) with partially characterized onconeural antibodies and no cancer. Partially characterized onconeural antibodies are infrequently seen in patients without cancer. Hence, the neurological syndromes that fulfill the above defined criteria should be considered possible PNS until the follow-up confirms or not the presence of an underlying tumor.

3. A non-classical neurological syndrome, no onconeural antibodies, and cancer present within two years of diagnosis: this definition may include neurological syndromes, in which we cannot rule out a casual association with as common an event as cancer.

To reduce false-positive diagnosis of possible PNS, the panel decided to limit the time period between the neurological syndrome and the diagnosis of cancer to two years. Although, some cases of sensorimotor neuropathy were considered potential PNS early in the description of these syndromes, they are clinically and neurophysiologically heterogeneous and are associated with various tumor types (12).

## Classical syndromes

The term ‘‘classical syndrome’’ applies to those neurological syndromes that frequently associate with cancer (Table 1). The diagnosis of a classical syndrome should prompt to investigate an occult tumor regardless of the antibody status. In adults, it has been estimated that about 3–5% of patients with small cell lung cancer, 15-20% with thymomas, and 3–10% with B-cell or plasma-cell neoplasms develop paraneoplastic neurologic disorders. The prevalence in cancer of the breast or ovary and other neoplasms is lower than 1% (13).

**Table 1:** Classical Paraneoplastic Neurological Syndromes

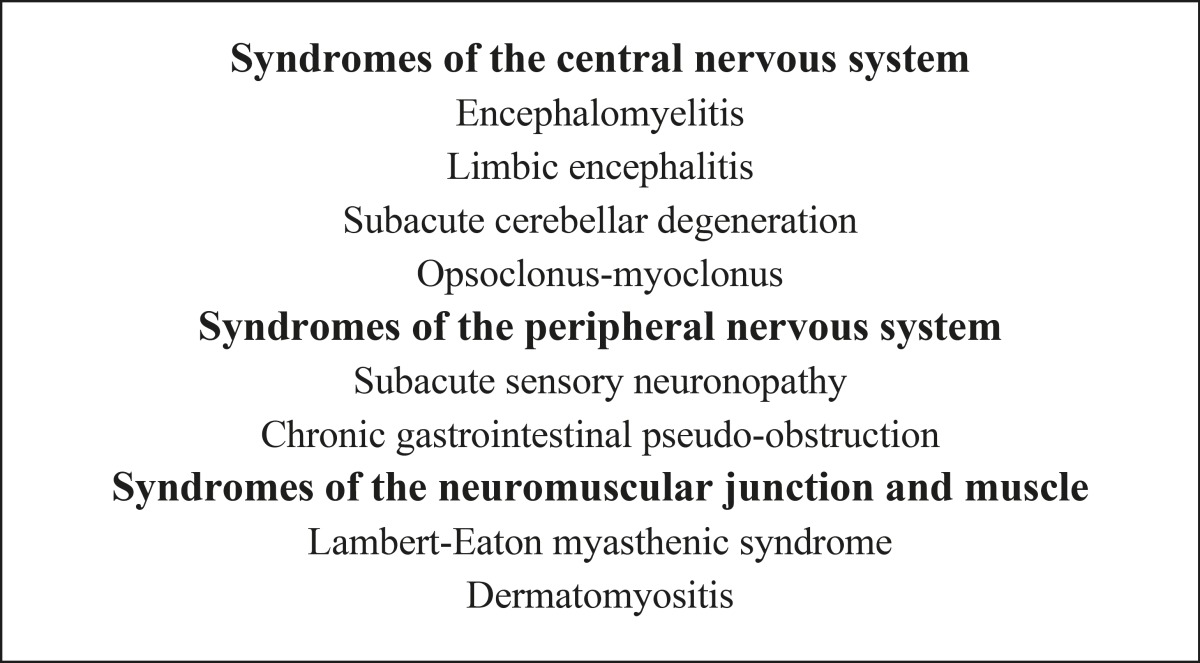

## Laboratory findings

Cerebrospinal fluid (CSF) shows a mild pleocytosis (30-40 WBC/mm3) and an elevated IgG level. Pleocytosis is usually seen only early in the course of the disease and disappears in several weeks to months. The elevated IgG1 may, however, persist. Analysis of CSF cells in patients with paraneoplastic cerebellar degeneration has shown that the predominant cell type (over 75%) is T cells, with a small component (less than 10%) of B cells and natural killer (NK) cells (14). Many patients with paraneoplastic syndromes have antibodies in their serum (and CSF), which react with both antigens in the nervous system and the underlying cancer. Although, there is considerable overlap, each of these antibodies is related to clinical syndromes and a restricted subgroup of cancers. These antibodies also suggest the location of the underlying cancer. For instance, the existence of anti- Yo antibodies in the serum of a woman with cerebellar symptoms can be a persuasive evidence that she has paraneoplastic cerebellar degeneration and ovarian cancer. Unfortunately, all patients with paraneoplastic syndromes do not have well-known antibodies in their serum. Whether this is a technical fault in detection or whether it means that some paraneoplastic neurologic disorders are not immune-mediated is not known (1). However, the antibodies and cytotoxic T-cells that are specific for the tumor antigen are not sufficient to cause the neurologic disease, unless they cross the blood– brain barrier and react with neurons that express the onconeural antigen (14).

## Well characterized onconeural antibodies

In the absence of a detected tumor, only well characterized onconeural antibodies (anti-Hu, Yo, CV2, Ri, Ma2, amphiphysin) should be applied for the classification of the associated disorder as definite PNS (Table 2). The term well characterized antibodies is based upon (1) antibodies for which there are recognizable patterns on routine immunohistochemistry and for which immunoblotting on recombinant proteins must be used for confirming their specificities (2); the number of cases reported to be associated with tumors (3); the description of well characterized neurological syndromes that are associated with the antibodies (4); the definite identification of the antibodies in different studies; and (5) frequency of these antibodies in patients without cancer (7).

## Partially characterized onconeural antibodies

Partially characterized onconeural antibodies include anti-Tr, ANNA3, PCA2, Zic4, and mGluR1. Other antineuronal antibodies occasionally detected in one or a few patients with PNS should not be utilized in the diagnosis of PNS until more data are obtained (8). Care must be taken to distinguish those that preferentially associate with PNS from those that may just reflect an antitumour immune response. Antibodies in paraneoplastic neurologic disorders react with the portion of the nervous system that accounts for the clinical symptoms, for example, anti Purkinje- cell antibodies occur in patients with paraneoplastic cerebellar degeneration (15). In paraneoplastic neurologic disorders that affect the brain, relatively high titers of the antibody in the CSF (relative to total IgG) show that the antibody is synthesized within the brain, probably by specific B cells that have crossed the blood-brain barrier (16). However, different paraneoplastic neurologic disorders may have various underlying mechanisms. When the target antigens are cell-surface receptors, like in the Lambert-Eaton myasthenic syndrome, myasthenia gravis, and a rare form of paraneoplastic cerebellar degeneration, antibodies can play a predominant role (1,14,16). In the current model, apoptosis of tumor cells triggers an antitumor immune response. Actually, apoptotic tumor cells in paraneoplastic neurologic disorders are strong stimulators of activating tumor-specific T cells. Such killer T cells can trigger a feedback loop via inducing apoptosis and therefore amplification of the antitumor immune response (17,18).

**Table 2:** Well Characterized Onconeuronal Antibodies and Related Tumors

Anti-Hu (ANNA1)	Small cell lung cancer
Anti-Yo (PCA1)	Ovary, breast
Anti-CV2 (CRMP5)	Small cell lung canc, thymoma
Anti-Ri (ANNA2)	Breast, Small cell lung cancer
Anti-Ma2 (Ta)	Testicular, lung
Anti-amphiphysin	Breast, Small cell lung cancer

## Paraneoplastic neurologic disorders in children

The general criteria for diagnosis of PNSs in adults can be applied to children. However, the maximal interval period between neurologic symptom presentation and demonstration of a tumor, that in adults has been suggested to be 5 years, should apparently be tailored to a shorter interval taking into account the tumors more frequently involved in children (neuroblastoma, teratoma, and Hodgkin’s lymphoma) (19). On the contrary, they sometimes mask the symptoms of a tumor and result in diagnostic delay (20). Although, paraneoplastic syndromes can affect any part of the neuraxis in children, the central nervous system is mostly affected. The prevalence of paraneoplastic neurologic disorders in children has not been known yet. The most common paraneoplastic syndromes of CNS in children comprise of opsoclonus myoclonus syndrome (OMS), limbic encephalitis, and Anti-N-methyl-D-aspartate receptor (anti-NMDA-R) encephalitis.

## Opsoclonus-myoclonus syndrome

OMS, also called Kinsbourne syndrome or “dancing eyes” syndrome, is the most common pediatric PNS in the medical literature. Patients may present with staggering and falling that often can lead to a misdiagnosis of acute cerebellar ataxia. Later, they may develop myoclonus, drooling, speech problems, hypotonia, and sleep disturbance (19). The duration of the time between presentation of neurologic symptoms and diagnosis of tumor in children varies between 1 week to 20 months after symptom onset (21). Most of the paraneoplastic syndromes in children are believed to be immune-mediated. The major explanation is that when proteins which are normally restricted to the nervous system become ectopically expressed in a cancer, the immune system recognizes the neural antigen (in the cancer) as foreign and elicits an attack, which may result in antineoplastic effects but also causes severe neurologic symptoms (19). In most cases, antibodies react with cell surface antigens and play a direct role in causing the neurologic disorders. Some of the PNSs seem to be mediated by a specific antigen-driven clonal expansion of T cells, in which T-cell immune responses are directed against the target antigens and finally, some cases of OMS may be both B-cell and T-cell mediated (13,19). In children, syndrome of opsoclonus, cerebellar ataxia, and myoclonus occurs mainly as a paraneoplastic syndrome related to occult, low-grade neuroblastoma (stage I or II). The syndrome occurs almost exclusively in young children (6 months to 3 years of age) and is generally considered to occur in about 2% to 3% of children with neuroblastoma (22,23). Survival in children with neuroblastoma presenting with opsoclonus-ataxia is significantly higher than in children with neuroblastoma without neurologic presentation (9,23).

## Antibody search

A paraneoplastic panel should be performed on the serum and possibly the CSF. If clinical suspicion is high, it is recommended that a tumor search be initiated at the time the antibody panel is sent. Antibodies do not have specificity and sensitivity in children, especially for neuroblastoma, and classic or atypical OMS. If paraneoplastic antibodies are present in association with these symptoms, the patient should be assumed to have an occult neoplasm until proven otherwise (21). In children with opsoclonus-ataxia, antineuronal antibodies are less consistently found and their related antigen is still unclear, although Connolly et al. reported antibodies to various components of cerebellar neurons in at least some children with opsoclonus-ataxia (24). Some researchers have found that serum autoantibodies against cell-surface antigens on cerebellar granular neurons and neuroblastoma cells, and incubation of these autoantibodies with neuroblastoma cell lines inhibits cell proliferation and induces apoptosis (25,26). However, these data could not be reproduced by other researchers using serum of patients with adult OMS (27).

## Tumor search

In general, a tumor search includes imaging of the neck, chest, abdomen, and pelvis, including testes in boys. A survey of OMS in UK showed wide variability in approaches, with MRI being more commonly done in children neuroblastoma. One fourth of neuroblastoma positive children initially had both a negative chest x-ray and abdominal ultrasound and the tumor was diagnosed with MRI (28). A UK report in 1994 on 54 patients with OMS showed neuroblastoma in only four patients, which increase the possibility that occult tumors were missed by the imaging modalities available at the time (29). After MRI, whole-body fluorodeoxyglucose– positron emission tomography (FDG-PET) may be needed to detect the tumor. In a study on 20 patients with paraneoplastic antibodies, in whom conventional imaging gave negative or uncertain results for the presence of tumor, abnormal uptake was found in 18 patients, 14 of whom had a histologically confirmed tumor (30). Pediatric neurologists may want to consider using FDG-PET more often, although no studies have compared FDG-PET to MRI for this intention. Lately, an European task force suggested thoracic CT followed by FDG-PET and pelvic ultrasound followed by CT. It is not obvious why MRI was not recommended. It is also recommended to repeat initial screening after 3-6 months and screening every 6 months up till 4 years (30). To search for neuroblastoma, tests should moreover include catecholamine metabolites (e.g., vanillylmandelic acid and homovanillic acid) in the urine, and nuclear scintigraphy studies using radioiodinated metaiodobenzylguanidine (MIBG) (31).

## Limbic encephalitis and anti - NMDA receptor encephalitis

Typical features that suggest involvement of the limbic system comprise personality changes, irritability, seizures, cognitive dysfunction, and memory loss. Retrospective studies indicate paraneoplastic syndromes may represent approximately 10% of pediatric limbic encephalitis cases (32). The most frequently associated neoplasms in children are Hodgkin lymphoma, ovarian teratoma, or testicular tumor. Neuroblastoma has also been reported (33). In adults, neurologic symptoms in paraneoplastic limbic encephalitis precede the cancer diagnosis in 60% of patients with a median of 3.5 months, and this number may be higher in children (34). The diagnosis of limbic encephalitis is clinical and its confirmation in adults requires CSF analysis, MRI demonstrating temporal lobe abnormalities, or electroencephalogram showing epileptic activity or slowing in the temporal lobes (34). There are no studies that examine the sensitivity and specificity of diagnostic methods in children with limbic encephalitis. Onconeuronal antibodies are detected in the serum and CSF of 60% of adult patients with paraneoplastic limbic encephalitis (36). Various antibodies and tumors have been reported, such as anti- Hu/ANNA-1 (small cell lung cancer), anti-Ri/ANNA-

2 (lung or breast cancer), anti-Ma2 (lung, breast, or testicular cancer), anti-CV2/CRMP5 (small cell lung cancer), and anti-amphiphysin (breast, small cell lung cancer) (34,35). Most antibodies and associated tumors are rare or not occurring in children.

## NMDA receptor antibodies

Anti - N - methyl - D - aspartate receptor (NMDAR) encephalitis is a recently described paraneoplastic syndrome that is increasingly recognized in children and adolescents. Since the first case reports in 2007 (36, 37) and a series in 2008 (38), hundreds of cases of paraneoplastic and non paraneoplastic anti- NMDAR encephalitis have been stated in the literature. Children with NMDAR encephalitis have prominent neuropsychiatric abnormalities, including behavioral or personality change, sleep dysfunction, dyskinesias or dystonias, autonomic instability, and speech reduction (39). Like other encephalitis, supporting evidence consists of CSF lymphocytic pleocytosis or oligoclonal bands demonstrating an inflammatory or immune- mediated process, EEG with slow, disorganized activity and infrequent seizures, and brain MRI that shows transient flair or contrast-enhancing abnormalities in cortical and subcortical regions, limbic areas, basal ganglia, brainstem, cerebellum, and sometimes white matter (39).

Other paraneoplastic neurologic syndromes in pediatrics Other cases of paraneoplastic syndromes have been reported in children. As in adults, there may be overlap among the various paraneoplastic syndromes. For example, it has been observed that patients with encephalitis have gastrointestinal dysmotility and sensory neuropathies (40). Hodgkin’s disease has been associated with paraneoplastic neurologic syndromes in children, including a case report of cerebellar degeneration and Horner syndrome (41). Children may have been found to have paraneoplastic disorders of neuromuscular transmission. Lambert- Eaton myasthenic syndrome characterized by muscle weakness, hyporeflexia, and autonomic dysfunction is associated with cancer in approximately 50% of cases, typically small cell lung cancer in adults and neuroblastoma in children (42).

## Treatment Overview

In any paraneoplastic syndrome in children, initial treatment must focus on the removal and treatment of the tumor. Almost all paraneoplastic syndromes have some improvement in neurologic symptoms following tumor removal (43). Most of the CNS paraneoplastic syndromes associated with antibodies against intracellular antigens have a poor response to treatment, while the childhood OMS and paraneoplastic syndromes associated with receptor/ion channel autoantibodies have a better response to immunosuppressive therapies, plasmapheresis, and intravenous immunoglobulins (IVIGs) (25). Possible treatment modalities are immunosuppression with corticosteroids or ACTH, plasma exchange, IVIG and chemotherapy, such as the alkylating agent cyclophosphamide, which acts as an immune suppressant or the anti-CD20 monoclonal antibody rituximab. In addition, supportive care and rehabilitation by speech therapy, physical therapy, and occupational therapy have an important role, as psychopharmacology and behavioral therapy do for emotional and behavioral problems. Although, these treatment modalities have not been systematically studied, they play a key role in the developing child. The best treatment for children with paraneoplastic OMS is still unknown. The traditional dose of prednisone has been 2 mg/kg/day with a prolonged taper, but recent reports have used monthly high-dose dexamethasone pulses of 20 mg/m2/day for 3 days (44). The length of treatment with steroids or ACTH varies with Pranzatelli et al. (45) that recommended a 52-week protocol. Immunosuppression with corticosteroids or ACTH often leads to improvement of acute OMS symptoms, and the preference of one over the other is a matter of debate. Improvement with rituximab has been reported in two patients with anti- Hu antibody associated encephalomyelitis (46). Alavi et al. reported a 3.5-year-old girl referred for ataxia and dancing eye movements starting since 1.5 years before who was diagnosed with neuroblastoma by imaging studies on admission. The OMS was refractory to surgical resection, chemotherapy, corticosteroids, and IVIG. Patient received rituximab (375 mg/m2/dose) simultaneously with chemotherapy. The total severity score decreased by 61.1% after rituximab therapy. Patient’s ataxia was markedly improved, so that she was able to walk independently after 6 months (47). Florance et al. found that 75% of adult and pediatric patients with anti-NMDR encephalitis had complete or considerable recovery following immunotherapy or tumor removal, while 25% of the patients were left with memory, cognitive, and motor deficits or died of the disorder (39). In 31 patients with paraneoplastic or non-paraneoplastic anti-NMDAR encephalitis, 30 (97%) had immunotherapy, including a combination of corticosteroids, IVIG, or plasma exchange (39).

Finally, it is important to emphasize that in about 20% of cases, no cancer is ever found, even at postmortem examination, probably indicating the successful control of tumor growth and metastasis by the host immune system in patients with araneoplastic neurologic disorder. Meanwhile, there are very rare associations of other conditions with malignancies in children that should not interpreted as paraneoplastic syndromes. Alavi et al. have reported a very rare occurrence of kawasaki disease concurrent with neuroblastoma (48), whereas it should not be interpreted as paraneoplastic syndrome.

**In conclusion, **PNSs are rare disorders so that even neurologists encounter only a few patients in their routine practice. The information collected from referring neurologists may be short of detailed clinical or paraclinical information, which may be important for a better definition of PNS. The diagnostic criteria presented in this review article may assist neurologists to report their patients with PNS more uniformly. The most important limitation in this field is the facilities to detect onconeural antibodies, since they have not been widely standardized, and with the discovery of new onconeural antibodies, care must be taken to differentiate those that associate with PNS from those that may just reflect an antitumour immune response. The future will provide evidence if these recommendations have been useful to improve the diagnostic accuracy and researches in the field of PNS.
